# Desiccation tolerance in resurrection plants: new insights from transcriptome, proteome and metabolome analysis

**DOI:** 10.3389/fpls.2013.00482

**Published:** 2013-11-28

**Authors:** Challabathula Dinakar, Dorothea Bartels

**Affiliations:** ^1^Institute of Molecular Physiology and Biotechnology of Plants, University of BonnBonn, Germany; ^2^Department of Life Sciences, School of Basic and Applied Sciences, Central University of Tamil NaduThiruvarur, India

**Keywords:** transcriptomics, proteomics, metabolomics, resurrection plants, desiccation tolerance

## Abstract

Most higher plants are unable to survive desiccation to an air-dried state. An exception is a small group of vascular angiosperm plants, termed resurrection plants. They have evolved unique mechanisms of desiccation tolerance and thus can tolerate severe water loss, and mostly adjust their water content with the relative humidity in the environment. Desiccation tolerance is a complex phenomenon and depends on the regulated expression of numerous genes during dehydration and subsequent rehydration. Most of the resurrection plants have a large genome and are difficult to transform which makes them unsuitable for genetic approaches. However, technical advances have made it possible to analyze changes in gene expression on a large-scale. These approaches together with comparative studies with non-desiccation tolerant plants provide novel insights into the molecular processes required for desiccation tolerance and will shed light on identification of orphan genes with unknown functions. Here, we review large-scale recent transcriptomic, proteomic, and metabolomic studies that have been performed in desiccation tolerant plants and discuss how these studies contribute to understanding the molecular basis of desiccation tolerance.

## INTRODUCTION

The sessile nature of plants has endowed them with a wide spectrum of adaptations to combat environmental perturbations. The mechanisms to survive under various environmental fluctuations are complex and vary widely. Drought, a physiological form of water stress or deficit, affects the performance of plants and leads to low crop yield. Most of the flowering angiosperm plants are drought sensitive and have relative water contents of around 85–100% under actively growing conditions and do not survive, if the water content falls below 59–30% (Höfler et al., 1941). In this context, *Arabidopsis thaliana* is considered as a model to study the responses of plants toward tolerating moderate water stress and to study the genes involved in this response. Although desiccation tolerance in seeds is common in higher plants, desiccation tolerance in vegetative tissues is restricted to the unique group of resurrection plants (Bartels and Hussain, 2011). Several resurrection species have been extensively studied for understanding the molecular basis of desiccation tolerance: the bryophyte *Tortula ruralis*, the clubmosses *Selaginella lepidophylla* and *Selaginella tamariscina*, the dicots *Craterostigma plantagineum*, *C. wilmsii*, *Boea hygrometrica*, and *Myrothamnus flabellifolia*, and the monocots *Xerophyta viscosa*, *X. humilis*, and *Sporobolus stapfianus* (Ingram and Bartels, 1996; Alpert and Oliver, 2002; Moore et al., 2009; Cushman and Oliver, 2011; Oliver et al., 2011a,b). The survival strategies often involve the re-activation of existing protection systems (Moore et al., 2009; Dinakar et al., 2012; Gechev et al., 2013). The importance of orphan genes/proteins/metabolites in the context of desiccation tolerance also needs to be considered. Most resurrection plants are polyploid with large genomes, difficult to transform and their genome sequences are not available; due to these features a mutational approach is at present not possible for functional analysis. Desiccation tolerance is controlled by many genes or proteins, therefore, a systems biology approach combining transcriptomics, proteomics, and metabolomics should be informative to understand the mechanism of desiccation tolerance, and to determine at which level of control the changes are affected. Here recent progress is reviewed on transcriptomic, proteomic, and metabolomic analyzes in resurrection plants and future prospects are discussed.

## “OMICS” APPROACHES TO UNDERSTAND DESICCATION TOLERANCE

Recent advances in “omics” technologies have enabled quantitative monitoring of the abundance of biological molecules in a high-throughput manner, thus making it possible to compare their levels between desiccation tolerant and sensitive species. Transcriptomic, proteomic, and metabolomic approaches attempt to capture complete information on the changes in transcripts/proteins/metabolites that take place during desiccation and subsequent rehydration thereby giving an outline of the metabolic situation. The identification of the abundant transcripts gives an indication which metabolic processes may be important at different physiological conditions.

## TRANSCRIPTOME ANALYSIS

Transcriptomics or mRNA expression profiling captures spatial and temporal gene expression and quantifies RNAs under different conditions. While quantitative analysis of gene expression can be done by either qRT-PCR (quantitative reverse transcriptase polymerase chain reaction) or by gene microarray, the most widely used early approach toward transcriptome analysis was the collection of expressed sequence tags (ESTs) which is limited to a few hundred or thousand sequenced cDNAs. Recent advances in sequencing technologies and assembly algorithms have facilitated the reconstruction of the entire transcriptome by deep RNA sequencing (RNA-seq), even without a reference genome, therefore, this is also applicable to resurrection plants (**Table 1**). Gene expression studies and EST sequencing have been performed in some resurrection species, such as the moss *T. ruralis* (Scott and Oliver, 1994; Wood and Oliver, 1999; Zeng et al., 2002; Oliver et al., 2004), the clubmosses *Selaginella lepidophylla* and *Selaginella tamariscina* (Zentella et al., 1999; Iturriaga et al., 2006; Liu et al., 2008), the monocot species *Sporobolus stapfianus* (Neale et al., 2000; Le et al., 2007), *X. viscosa* (Mundree et al., 2000; Mowla et al., 2002; Lehner et al., 2008), *X. humilis* (Collett et al., 2003, 2004; Illing et al., 2005; Mulako et al., 2008), and *X. villosa* (Collett et al., 2004), and the dicot species *C. plantagineum* (Bockel et al., 1998). In these studies, the cDNA libraries for EST sequencing were either generated from one or two physiological conditions (dehydrated and rehydrated gametophytes/fronds/roots or leaves) and restricted in number thereby not always reflecting global transcript changes. Comprehensive transcriptome analysis have so far been reported for *C. plantagineum* and *Haberlea rhodopensis* (Rodriguez et al., 2010; Gechev et al., 2013).

**Table 1:** Omics studies carried out in resurrection plants and sister group comparisons between desiccation tolerant and sensitive plants.

Approach	Desiccation tolerant species	Desiccation sensitive species	Reference
**Resurrection plants**
Transcriptome analysis	*Craterostigma plantagineum*		Rodriguez et al. (2010)
Transcriptome and metabolomic analysis	*Haberlea rhodopensis*		Gechev et al. (2013)
Proteome analysis	*Selaginella tamariscina*		Wang et al. (2010)
Proteome analysis	*Xerophyta viscosa*		Ingle et al. (2007)
Proteome analysis	*Boea hygrometrica*		Jiang et al. (2007)
Proteome analysis	*Sporobolus stapfianus*		Oliver et al. (2011a)
Metabolomic analysis	*Selaginella lepidophylla*		Yobi et al. (2013)
**Sister group comparisons**
Metabolomic comparison	*Sporobolus stapfianus*	*Sporobolus pyramidalis*	Oliver et al. (2011b)
EST sequencing and comparison	*Selaginella lepidophylla*	*Selaginella moellendorffii*	Iturriaga et al. (2006)
Metabolomic comparison	*Selaginella lepidophylla*	*Selaginella moellendorffii*	Yobi et al. (2012)

Transcriptome sequencing of *C. plantagineum* and *H. rhodopensis* at four different physiological stages (control, partially dehydrated, desiccated, and rehydrated) revealed that the overall identified transcripts have highest similarity to genes of *Vitis vinifera*, *Ricinus communis*, and *Populus trichocarpa.* In *C. plantagineum*, 182 MB of transcript sequences were assembled into 29,000 contigs which yielded more than 15,000 uniprot identities and in *H. rhodopensis* 96,353 expressed transcript contigs were identified (Rodriguez et al., 2010; Gechev et al., 2013). EST sequencing of a cDNA library from the rehydrated moss *T. ruralis* resulted in the characterization of around 10,368 ESTs representing 5,563 genes (Oliver et al., 2004). An interesting feature that resulted from these studies is that about one-third of the contigs from *C. plantagineum* and around 40% of the sequences from *H. rhodopensis* and *T. ruralis* did not map to uniprot identities and encode unknown transcripts which are potential sources for gene discovery. The transcripts can be divided into two main groups according to expression patterns observed for *C. plantagineum* and *H. rhodopensis*. The first group consists of transcripts abundant in control and rehydrated tissues and the second group consists of transcripts abundant in dehydrated and desiccated tissues (Rodriguez et al., 2010; Gechev et al., 2013).

### TRANSCRIPTS ABUNDANTLY EXPRESSED IN FULLY HYDRATED TISSUES AND UNDER REHYDRATION CONDITIONS

The most abundant transcripts in fully hydrated and rehydrated conditions encode proteins involved in photosynthesis and carbohydrate metabolism. In *C. plantagineum* and *H. rhodopensis*, the transcripts encoding RuBisCO activase, carbonic anhydrases, fructose bisphosphatases, chlorophyll a/b binding protein, light harvesting complex, and RuBisCO small subunit are highly abundant in fully hydrated conditions. The transcripts related to photosynthesis decline gradually upon dehydration suggesting decreasing photosynthetic activity as the primary target during dehydration (Rodriguez et al., 2010; Gechev et al., 2013). A similar trend in the gene expression related to photosynthesis was also observed in *X. viscosa* (Collett et al., 2003).

Comparison of genes abundantly expressed in fully hydrated conditions indicates the presence of different pathways in *C. plantagineum* and *H. rhodopensis*. Galactinol synthases which catalyze the first step in the synthesis of raffinose oligosaccharides are abundantly expressed in fully hydrated leaves of *C. plantagineum*, but in *H. rhodopensis* their expression is high during dehydration and desiccation suggesting that raffinose synthesis is induced in *H. rhodopensis* at later stages of dehydration (Rodriguez et al., 2010; Gechev et al., 2013). Other abundant transcripts in *C. plantagineum* encode acid phosphatases that are required for the maintenance of cellular inorganic phosphate levels. The transcripts encoding proteins involved in ion transport such as membrane-associated carriers together with proteins involved in cell wall plasticity and membrane integrity such as xyloglucan endotransglucosylases and members of the expansin gene family are abundant in fully hydrated conditions in *C. plantagineum* (Rodriguez et al., 2010). In *H. rhodopensis* two of the most abundant transcripts observed in hydrated conditions are catalase encoding genes that are not observed in *C. plantagineum* (Rodriguez et al., 2010; Gechev et al., 2013).

A feature that was observed in rehydrated *H. rhodopensis* plants is the abundance of genes encoding an auxin efflux carrier, hypothetical proteins and genes with unknown functions (Gechev et al., 2013). In *C. plantagineum*, the abundant transcripts in rehydrated tissues are related to defense, oxidative stress, and metabolism of vitamin K-related compounds (Rodriguez et al., 2010). Several of the transcripts expressed under rehydration conditions in *C. plantagineum* encode pathogen responsive proteins, carbohydrate metabolism associated enzymes like transketolases, enzymes related to phylloquinone metabolism which are electron transfer cofactors in photosystems, and peroxidase transcripts which function in detoxification of reactive oxygen species (ROS; Rodriguez et al., 2010).

### TRANSCRIPTS INDUCED IN MILDLY DEHYDRATED AND DESICCATED TISSUES

Generally genes induced during dehydration code for proteins that prevent stress-related cellular damage and participate in antioxidant defense. Dehydration-induced transcripts encode proteins with protective properties, enzymes related to carbohydrate metabolism, regulatory proteins such as transcription factors, kinases, and signaling molecules as well as unknown proteins (Iturriaga et al., 2006; Rodriguez et al., 2010; Gechev et al., 2013).

### TRANSCRIPTS ENCODING LATE EMBRYOGENESIS ABUNDANT PROTEINS

Late embryogenesis abundant (LEA) genes comprise the most abundantly up-regulated group of genes in response to dehydration. In *H. rhodopensis* some LEA genes are constitutively expressed in hydrated conditions and their expression is induced to higher levels upon drought and desiccation suggesting that the transcriptome of *Haberlea* is primed already for dehydration and desiccation tolerance (Gechev et al., 2013). Genes encoding LEA proteins are abundant in dehydrated leaves of *C. plantagineum* but unlike *Haberlea* not in fully hydrated tissues (Piatkowski et al., 1990; Michel et al., 1994; Rodriguez et al., 2010). LEA proteins are localized in different cellular compartments such as cytosol, chloroplasts, mitochondria, or nuclei. Dehydration-induced expression of LEA transcripts is a common response in resurrection plants as well as in desiccation sensitive plants (Bartels et al., 1990; Collett et al., 2004; Oliver et al., 2004; Rodriguez et al., 2010; Gechev et al., 2012, 2013). The major difference between desiccation tolerant and sensitive plants seems to be in the abundance of the transcripts. As an example, the LEA-like CDeT11-24 transcript is highly expressed during desiccation in *C. plantagineum*, whereas the transcript is expressed at a low level in desiccation sensitive *Lindernia subracemosa* plants. This suggests that the CDeT11-24 transcript in *L. subracemosa* is unstable or induced at a lower rate during dehydration. Comparative promoter analysis of the CDeT11-24 gene between *C. plantagineum* and *L. brevidens* showed different promoter activities and the absence of dehydration specific promoter *cis* elements in *L. brevidens*, which correlates with the transcript expression level (van den Dries et al., 2011). These results lead to the hypothesis that high level gene expression under dehydration is evolved by selection of promoter *cis* elements.

### TRANSCRIPTS ENCODING PROTEINS RELATED TO DETOXIFICATION AND ANTIOXIDANT DEFENSE

Reactive oxygen species such as superoxide, hydrogen peroxide, and hydroxyl radicals are unavoidable by-products of aerobic metabolism and are commonly generated during dehydration stress. Since ROS can potentially damage proteins, lipids, and nucleic acids, genes encoding antioxidant enzymes are supposed to be up-regulated in resurrection plants. Transcriptome analysis confirmed an up-regulation of genes involved in antioxidative defense. In hydrated conditions resurrection plants maintain high levels of antioxidants, which increase upon stress. This feature is observed in all resurrection species studied so far (Sherwin and Farrant, 1998; Farrant, 2000; Kranner et al., 2002). The transcriptome of *H. rhodopensis* contained an extensive antioxidant gene network in the fully hydrated state. The number of expressed genes encoding superoxide dismutases, catalases, monodehydroascorbate reductases, and glutathione (GSH) reductases are higher in *H. rhodopensis* than in *C. plantagineum* in the hydrated state. Expression of these genes is even further up-regulated during dehydration in *H. rhodopensis* (Gechev et al., 2013). Induction of genes related to the antioxidant pathway during desiccation has also been reported in the desiccation tolerant plant *Sporobolus stapfianus* (Neale et al., 2000). In *X. humilis*, a plant that looses chlorophyll during desiccation, also a large number of antioxidant defense genes are up-regulated during dehydration (Collett et al., 2004). Besides conserved antioxidant genes some resurrection plants acquired expression of genes from other pathways. An example was reported for *X. viscosa*, in which the desiccation-induced antioxidant gene encoding 1-Cys peroxiredoxin (XvPer1) shows 70% sequence identity to *Arabidopsis* seed specific dormancy related 1-Cys peroxiredoxin (AtPer1; Haslekás et al., 1998; Ndima et al., 2001; Mowla et al., 2002). In the *C. plantagineum* transcriptome analysis thiamine biosynthesis transcripts and aldehyde dehydrogenase (CpALDH) transcripts are up-regulated during dehydration and contribute to antioxidant defense (Rodriguez et al., 2010). In *T. ruralis* the transcript levels of an aldehyde dehydrogenase (ALDH21A1) were also increased during dehydration suggesting ALDH as a stress regulated enzyme with the potential to detoxify excess amounts of aldehydes (Chen et al., 2002; Stiti et al., 2011). Transgenic *Arabidopsis* plants over-expressing AtALDH3 showed tolerance to dehydration stress and low accumulation of malondialdehyde thereby emphasizing the importance of aldehyde dehydrogenase in conferring tolerance to oxidative stress (Sunkar et al., 2003).

Another group of transcripts that are induced during dehydration/desiccation encode early light induced proteins (ELIPs). These ELIPs are nuclear-encoded proteins associated with thylakoid membranes in the chloroplast and are believed to bind to chlorophyll thus preventing ROS-induced photooxidative damage. Several desiccation-related genes encoding ELIPs have been isolated from *C. plantagineum*. DSP-22 a homolog of a class of ELIPs is thought to stabilize photosynthetic structures within the chloroplasts of *C. plantagineum* and ameliorate rehydration-induced damage (Bartels et al., 1992; Ingram and Bartels, 1996). In *C. plantagineum*, the dsp-22 transcript is undetected in unstressed plants but abundantly expressed during desiccation in light (Alamillo and Bartels, 1996). ELIPs are also abundantly expressed during dehydration in *H. rhodopensis* (Gechev et al., 2013). In *Sporobolus stapfianus* induction of transcripts encoding ELIPs was observed only in desiccation tolerant tissues but not in desiccation sensitive tissues, which supports the role for ELIPs in desiccation tolerance (Neale et al., 2000).

### TRANSCRIPTS ENCODING ENZYMES RELATED TO CARBOHYDRATE METABOLISM

Accumulation of sucrose and raffinose oligosaccharides is commonly observed in resurrection plants (Bartels and Hussain, 2011). Their accumulation correlates with the up-regulation of transcripts encoding enzymes of carbohydrate metabolism. In *C. plantagineum* and *H. rhodopensis*, several genes encoding galactinol synthases and a stachyose synthase are present in hydrated leaves and are additionally up-regulated during dehydration (Rodriguez et al., 2010; Gechev et al., 2013) suggesting the importance of oligosaccharides in protecting cells during desiccation. Similarly, the cDNAs encoding enzymes of the polyol biosynthesis and raffinose family oligosaccharides are abundantly expressed upon dehydration in *X. humilis* (Illing et al., 2005). A high induction is observed for several sucrose synthases, sucrose 6-phosphate synthases, a sucrose transporter, and a sucrose responsive element-binding protein in *H. rhodopensis* during dehydration and desiccation (Gechev et al., 2013). Induction of transcripts encoding sucrose synthases and sucrose-phosphate synthases is also observed in *C. plantagineum* during dehydration (Ingram and Bartels, 1996; Kleines et al., 1999). The role of sugar metabolism for the adaptation of *C. plantagineum* and *H. rhodopensis* to desiccation is further substantiated by the presence of transketolase transcripts (*tkt*). Transketolases are key enzymes of the reductive and oxidative pentose phosphate pathways that are responsible for the synthesis of sugar phosphate intermediates, which can flow into different pathways. *C. plantagineum* has three transketolase isoforms (*tkt3*, *tkt7*, *tkt10*) and it has been suggested that a transketolase isoform is involved in octulose synthesis (Willige et al., 2009). While *tkt7* is more abundant in rehydrating tissues of *C. plantagineum*, *tkt10* is preferentially expressed in fully hydrated tissues. *Tkt3* is constitutively expressed and is involved in the Calvin cycle (Bernacchia et al., 1995; Rodriguez et al., 2010). Compared to *C. plantagineum*, two *tkt* are observed in *H. rhodopensis*, which differs in its carbohydrate metabolism from *C. plantagineum* (Gechev et al., 2013).

### TRANSCRIPTS RELATED TO CELL WALL MODIFICATION

Several reversible modifications occur to stabilize cell wall architecture in resurrection plants, while some of these changes are constitutive some are inducible. Cell wall folding plays an important role in mechanical stabilization during desiccation (Farrant et al., 2007). The modifications in cell wall properties to resist the mechanical stress during dehydration in resurrection plants have been discussed by Vicré et al. (2004) and Moore et al. (2006, 2008, 2013). Plasticity of cell walls is particularly important to avoid damage due to mechanical stress imposed during desiccation and rehydration (Moore et al., 2013). Transcripts encoding proteins involved in the maintenance of cell wall plasticity such as xyloglucan endotransglucosylases are abundantly expressed in unstressed tissues of *C. plantagineum* (Rodriguez et al., 2010). Concomitant with the increased cell wall extensibility transcripts encoding alpha expansins are up-regulated during desiccation in *C. plantagineum* (Jones and McQueen-Mason, 2004). In *H. rhodopensis*, genes encoding xyloglucan endotransglucosylases, pectin esterases, and pectate lyases are abundantly expressed in hydrated conditions and are switched off during dehydration. Concomitantly, laccase genes involved in lignin biosynthesis are only expressed in desiccated tissues pointing toward cell wall remodeling during desiccation (Gechev et al., 2013).

### TRANSCRIPTS ENCODING REGULATORY MOLECULES AND PARTICIPATING IN GENERAL METABOLISM ARE UP-REGULATED DURING DEHYDRATION

The transcriptome of desiccated tissues also includes genes that are either involved in general metabolism or that are related to environmental stresses other than dehydration. Examples are temperature-induced lipocalins, aquaporins, tonoplast intrinsic proteins, cation transporters, and rare cold inducible 2A protein. Lipocalins are membrane-associated proteins with a low-temperature response element, dehydration-responsive elements, and heat shock elements in their promoters suggesting their involvement in various abiotic stress conditions. A YSIRK signaling peptide, an alcohol oxidase, and genes encoding heat shock proteins are expressed in desiccated *H. rhodopensis* leaves. The occurrence of these types of transcripts implies that these genes have become dehydration-responsive during evolution.

The massive expression of transcripts during dehydration and the re-synthesis during rehydration requires a fine-tuned regulatory network. This is reflected by the fact that transcripts with a regulatory function comprise a large heterogenous group. Transcripts of desiccated *C. plantagineum* are dominated by those encoding DNA binding proteins, cysteine proteases, and proteins of amino acid metabolism. These transcripts are mostly members of gene families which participate in diverse pathways. During evolution some family members have acquired regulatory *cis* elements which trigger their expression in response to dehydration. In *H. rhodopensis* induction in gene expression upon dehydration was observed for a gene encoding a putative protein phosphatase/hydrolase which was not detected in hydrated/rehydrated samples indicating the importance of phosphorylation and dephosphorylation during dehydration (Gechev et al., 2013). Genes coding for transcription factors, heat shock proteins, and components of signaling cascades are among the transcripts expressed in response to dehydration in all resurrection plants. In the transcriptome of *H. rhodopensis*, a broad range of transcription factors have been identified such as MYB, NAC, WRKY, GRAS family members, DREB2, NF-YA, MADS-box transcription factors, and several heat shock transcription factors (Gechev et al., 2013). Some of the regulatory transcripts are exclusively expressed in desiccated samples, e.g., a receptor like protein kinase, kinases, phosphatases, a Ca^2^^+^ antiporter cation exchanger, and a phospholipase D isoform.

The few examples cited above show that the transcriptome analysis provides a catalog of the regulatory genes that are up-regulated during dehydration, but at present it is not understood how these genes interact with other pathways and which target genes they regulate.

## PROTEOME ANALYSIS

Translational regulation of mRNA is an important step in the control of gene expression. Changes in gene expression at the transcript level need not always correspond to changes in the protein level due to either transcript instability or post-transcriptional modifications. Post-translational modifications and protein degradation modulate the quality and quantity of expressed proteins and thus affect the correlation of transcript and protein levels. The main limitation of proteomics is the identification of the proteins due to absence of genome sequence information in resurrection plants. Therefore, functions have to be attributed according to homologies. Reports on proteome analysis in resurrection plants are restricted to a few species. A direct correlation between transcript and protein abundance was observed for many of the dehydration-induced gene products in particular for gene products with protective functions (Ingle et al., 2007; Jiang et al., 2007; Wang et al., 2010; Oliver et al., 2011a).

Qualitatively proteome data correlate with transcript data and confirm that the abundant proteins in the hydrated tissues are related to photosynthesis and carbohydrate metabolism. Proteome data demonstrated that the deactivation of photosynthetic activity and subsequent re-activation are major responses observed upon sensing dehydration and after rewatering, respectively (Ingle et al., 2007; Rodriguez et al., 2010; Wang et al., 2010; Oliver et al., 2011a). The decline of photosynthesis coincided with the decrease in chloroplast-localized photosynthetic proteins such as psbO, psbP (the two components of luminal oxygen evolving complex of PSII), the PSII stability factor HCF136, the α subunit of the F-ATPase, and the Calvin cycle enzyme transketolase in *X. viscosa* during dehydration at 35% relative water content (Ingle et al., 2007). Similarly photosynthesis-related proteins that decreased in abundance in *Selaginella tamariscina* during dehydration included RuBisCO large sub unit, chlorophyll *a*/*b* binding protein, and oxygen evolving complex protein (Wang et al., 2010).

During dehydration, LEA proteins accumulate abundantly in resurrection plants supporting their protective roles (Michel et al., 1994; Velasco et al., 1994; Alamillo and Bartels, 1996; Ingram and Bartels, 1996; Ndima et al., 2001). Using two dimensional SDS-PAGE coupled with a phosphoprotein specific stain at least two LEA proteins CDeT11-24 and CDeT6-19 were shown to be phosphorylated in *C. plantagineum* (Röhrig et al., 2006). Although the role of the protein phosphorylation in these two proteins is still unclear, phosphorylation may increase the hydrophilic residues necessary for interaction with other macromolecules or phosphorylation may be required for correct subcellular localization, as it was shown for maize embryo LEA proteins (Goday et al., 1988).

Proteome analysis also revealed the expression of unknown proteins in resurrection plants. In *Selaginella tamariscina*, 138 dehydration-responsive protein spots representing 103 unique proteins with unknown functions were identified (Wang et al., 2010). The proteins down-regulated in *Selaginella tamariscina* during dehydration included proteins involved in photosynthesis, carbohydrate and energy metabolism, stress and defense proteins, signaling, membrane transport, cell structure, and cell division. The protein abundance increased for antioxidant enzymes (Wang et al., 2010). From *B. hygrometrica* leaves more than 200 proteins were analyzed out of which 78 (35%) increased in expression in response to dehydration and 5% were induced in rehydrated leaves and 60% showed decreased or unchanged levels (Jiang et al., 2007). Several of the proteins related to antioxidant and energy metabolism are constitutively expressed, indicating that protective mechanisms exist constitutively which emphasize the preparedness of the plant for stress. Dehydration-induced proteins in *B. hygrometrica* are associated with energy metabolism, GSH and polyphenol metabolism. This indicates that GSH may serve as a major antioxidant in *B. hygrometrica*. Protein analysis also indicated degradation of photosynthesis-related proteins. A 20-kDa fragment of the RuBisCO large subunit (RbcL) and a 23-kDa polypeptide of the oxygen evolving complex of photosystem II were identified in dehydrated leaf proteins. The appearance of the 20-kDa RbcL protein fragment in *B. hygrometrica* is thought to be the result of stress-induced proteolysis mediated through ROS-induced chloroplast-localized protease activity (Jiang et al., 2007). ABC transporters that mediate ATP-dependent transport of solutes were also induced during dehydration in *B. hygrometrica* (Jiang et al., 2007). The induction of putative ATPase subunits matching a vacuolar H^+^-ATPase A subunit during dehydration may help in preparation for rehydration. Desiccated leaves of *Sporobolus stapfianus* and *X. viscosa* showed similar protein profiles as *B. hygrometrica* (Blomstedt et al., 1998; Marais et al., 2004). Enzymes related to sugar metabolism, such as sucrose synthase, ADP-glucose pyrophosphorylase, and GDP-mannose 3,5-epimerase were up-regulated during dehydration confirming the importance of sugar metabolism.

The protein expression patterns observed in different resurrection plants lead to the conclusion that stress protective proteins are rapidly and massively induced upon dehydration and present throughout desiccation. The induced proteins are involved in diverse functions such as scavenging ROS, accumulation of sucrose, protective proteins, cell wall remodeling proteins, and proteins with unknown functions.

There are examples in which mRNA levels do not correlate with protein expression patterns. Although the transcript levels of *tkt3* are constitutively expressed in *C. plantagineum* vegetative tissues, the protein levels are higher in hydrated tissues, which suggest a high translation rate or slower protein turnover during hydrated conditions. Similarly, the abundance of *tkt7* mRNA during late phases of rehydration in *C. plantagineum* does not match the protein abundance (Bernacchia et al., 1995). This may also be true for regulatory genes like transcription factors, which are often difficult to investigate due to the low abundance of these proteins.

## ENZYME ACTIVITIES

Many of the stress-induced proteins are enzymes and thus measuring their enzyme activities indicates whether their activities are maintained despite dehydration. Enzymes such as those involved in antioxidant synthesis, carbohydrate and nitrogen metabolism showed high enzymatic activities during dehydration/desiccation. This confirms that the protein activities are not affected by dehydration and are protected, e.g., by LEA proteins (Illing et al., 2005; Farrant et al., 2007; Petersen et al., 2012). In *X. viscosa*, the activities of ascorbate peroxidase, GSH reductase, and superoxide dismutase increased during dehydration and declined during rehydration whereas in *C. wilmsii* the activities of GSH reductase and superoxide dismutase increased upon rehydration (Sherwin and Farrant, 1998) indicating diversity of defense pathways in the poikilochlorophyllous monocot plant and the homoiochlorophyllous dicot plant.

Sucrose accumulation correlates with the up-regulation of carbohydrate metabolic enzymes in desiccated tissues. During dehydration, increased hexokinase activity is correlated with sucrose accumulation and the decline in glucose and fructose levels in both *Sporobolus stapfianus* and *X. viscosa* (Whittaker et al., 2001). Sucrose-phosphate synthase activity in leaves of *C. plantagineum* and *Sporobolus stapfianus* increases along with sucrose accumulation during dehydration indicating the redirection of carbon flow to sucrose from reserve substances such as starch and octulose (Whittaker et al., 2007). In *Sporobolus stapfianus*, increases in hexose sugars, sucrose, and amino acids are associated with concomitant increases in sucrose-phosphate synthase and pyruvate kinase (PK) activities, and maximal activity levels of phosphoenolpyruvate carboxylase (PEPCase), NADP-dependent isocitrate dehydrogenase (NADP-ICDH), and NADH-dependent glutamate synthase (NADH-GOGAT; Whittaker et al., 2007).

## METABOLOMIC ANALYSIS

Metabolomic approaches deal with quantitative analysis of small molecules giving a detailed analysis of the plant’s metabolic state. Because of the large variety of chemical structures and the different biochemical properties of molecules, no single technique and no single metabolite extraction protocol will identify all metabolites in a plant cell. Several approaches including gas chromatography–mass spectrometry (GC–MS), liquid chromatography (LC)–MS, capillary electrophoresis (CE)–MS, and nuclear magnetic resonance (NMR) spectroscopy are commonly used in plant metabolomics research. Although large-scale metabolomic studies have been reported for *Arabidopsis* plants undergoing dehydration (Urano et al., 2009), metabolomic studies have so far only been reported for two closely related *Sporobolus* and *Selaginella* species which differ in desiccation tolerance (Oliver et al., 2011b; Yobi et al., 2012). Metabolite changes during dehydration/rehydration include carbohydrates, amino acids, nucleotide derivatives, lipids, polyamines, antioxidants, and defense compounds. Several key metabolites have been identified from the metabolome studies in resurrection plants and are described below.

The metabolic profile of fully hydrated *Selaginella lepidophylla* is distinct from the dehydration state. The mapping of the known metabolites (66.5%) into the general biochemical pathways revealed that the more prevalent metabolites are amino acids (19%) followed by carbohydrates, lipids, cofactors, nucleotides, peptides, and secondary metabolites. While the amino acids, peptides, and nucleotide metabolites are overrepresented during desiccation, carbohydrates such as 4C–6C containing sugars, sugar alcohols, lipids, or lipid metabolites (with the exception of choline phosphate), and cofactors are overrepresented in the hydrated state (Yobi et al., 2013). Besides the known 251 metabolites that were identified in *Selaginella lepidophylla*, 33.4% (84) represented unknown metabolites. Seven of the unknown metabolites showed greater abundance under dehydration conditions than in hydrated conditions suggesting their role in desiccation tolerance. *Sporobolus stapfianus* is metabolically primed for dehydration as it contains high concentrations of osmolytes and nitrogen metabolites along with low levels of metabolites associated with energy metabolism in the hydrated state. Upon dehydration, the metabolism is shifted toward antioxidant production, carbohydrate production, nitrogen remobilization, and ammonia detoxification (Oliver et al., 2011b). This appears to be different in *C. plantagineum* where major metabolite changes are induced by dehydration (see below).

### CARBOHYDRATES

Carbohydrate metabolism plays a critical role in cellular protection in resurrection plants. Some carbohydrate metabolites change drastically in abundance during dehydration and rehydration. **Table 2** displays a summary of the main carbohydrate changes observed in various resurrection plants. Accumulation of sucrose, trehalose, and short-chain oligosaccharides such as raffinose has been observed during dehydration (Bianchi et al., 1991b; Drennan et al., 1993; Norwood et al., 2000; Peters et al., 2007; **Table 2**). Sucrose is the major carbohydrate in tissues of angiosperm resurrection plants upon desiccation (Bianchi et al., 1991b; Ghasempour et al., 1998; Norwood et al., 2000; Scott, 2000; Whittaker et al., 2001; Cooper and Farrant, 2002; Zivkovic et al., 2005). The importance of sucrose is demonstrated in a comparative metabolic analysis of desiccation tolerant *Eragrostis nindensis* and desiccation sensitive *Eragrostis* species. While *E. nindensis* accumulates sucrose in desiccated leaves, desiccation sensitive species of *Eragrostis* do not accumulate sucrose (Illing et al., 2005). Although trehalose is a critical key compound of some lower order desiccation tolerant organisms and yeast, it is a minor component in a few desiccation tolerant angiosperm species (Ghasempour et al., 1998).

**Table 2:** Carbohydrate metabolites in hydrated and dehydrated/desiccated conditions in vegetative tissues of resurrection plants.

Species	Hydrated	Dehydrated/desiccated	Reference
*Craterostigma plantagineum*	2-Octulose	Sucrose	Bianchi et al. (1991b)
*Craterostigma wilmsii*			Cooper and Farrant (2002)
*Haberlea rhodopensis*	Sucrose, raffinose	Sucrose, raffinose maltose, verbascose, stachyose	Djilianov et al. (2011), Gechev et al. (2013)
*Myrothamnus flabellifolia*	Fructose, glucose	Sucrose, arbutin, glucosylglycerol	Bianchi et al. (1993)
*Boea hygroscopica*	Glucose, fructose, sucrose, inositol	Sucrose	Bianchi et al. (1991a)
*Sporobolus stapfianus*	Glucose, fructose, sucrose	Sucrose, raffinose, stachyose	Whittaker et al. (2001), Oliver et al. (2011b)
*Xerophyta viscosa*	Galactinol, myo-inositol	Sucrose, raffinose	Peters et al. (2007)
*Selaginella lepidophylla*	Trehalose, sucrose, and glucose	Trehalose, sucrose, glucose	Yobi et al. (2013)

In *C. plantagineum* and *C. wilmsii* an extremely high concentration of the unusual C8 sugar, 2-octulose accumulates in well watered leaves (Bianchi et al., 1992; Norwood et al., 2000; Cooper and Farrant, 2002) and upon dehydration, the level of this sugar declines and the concentration of sucrose increases (Bianchi et al., 1991b). This conversion accounts for up to 80% of the carbohydrates in desiccated leaves of *Craterostigma*. According to Norwood et al. (2000), 2-octulose acts as a temporary storage carbohydrate like starch that accumulates in leaves during active photosynthesis and is mobilized at night. *C. plantagineum* plants seem to depend on the available carbohydrate source (2-octulose) for sucrose accumulation rather than relying on photosynthesis. Although *C. plantagineum* plants synthesize starch during photosynthesis, the amount of carbon entering the starch for storage compared to 2-octulose is small (Norwood et al., 2000).

In *Sporobolus stapfianus*, equimolar levels of fructose and glucose are present in fully hydrated tissues, dehydration causes a rapid increase in glucose levels thereby increasing the glucose to fructose ratio (Whittaker et al., 2001), which may be indicative of amylolytic starch breakdown during mild dehydration. However, upon desiccation both glucose and fructose levels decreased concomitant with an increase in sucrose (Ghasempour et al., 1998). Apart from high accumulation of sucrose and raffinose, stachyose, maltotetraose, and myo-inositol have also been reported to accumulate during desiccation. Maltotetraose and myo-inositol are likely to be synthesized from glucose-6-phosphate that stores phosphate during dehydration (Oliver et al., 2011b).

In *H. rhodopensis*, constitutive high accumulation of sucrose and raffinose seems to be an adaptive feature to survive dehydration. This supports the notion that *H. rhodopensis* is primed for desiccation as it was observed for antioxidants (Djilianov et al., 2011). Upon dehydration sucrose increases to even higher levels together with an increase in maltose, verbascose, and stachyose (Djilianov et al., 2011; Gechev et al., 2013).

The shrub *M. flabellifolia* synthesizes some interesting carbohydrates and the uncovering of the biosynthetic pathways of these carbohydrates could be utilized for the production of these substances *in vitro*. Besides sucrose, the glucoside arbutin, and glucosylglycerol (glucopyranosyl-β-glycerol) are synthesized during dehydration whereas fructose and glucose are decreased (Bianchi et al., 1993). Trehalose is present in both hydrated and dehydrated conditions. Glucosylglycerol acts as an osmoprotectant in cyanobacteria (Ferjani et al., 2003). Arbutin possesses anti-parasitic properties toward fungal and herbivore attack. The presence of the arbutin, glucosylglycerol, and trehalose appear to be specific for *M. flabellifolia*.

The carbohydrate profile of *Selaginella lepidophylla* resembles lower organisms like yeast. In *Selaginella lepidophylla*, trehalose, sucrose, and glucose are the most abundant metabolites and account for 50% of the total metabolites. The relative level of trehalose is higher than sucrose and glucose and does not change upon desiccation (Yobi et al., 2013). Several sugar alcohols such as sorbitol, xylitol, arabitol, erythritol, myo-inositol, and mannitol are abundant in *Selaginella lepidophylla* under hydrated conditions. They have been suggested to act as osmoprotectants by stabilizing protein structures against denaturation (Yobi et al., 2013). In *Selaginella lepidophylla*, many of the glycolysis/gluconeogenesis intermediates (glucose-6-phosphate, fructose-6-phosphate, maltose-6-phosphate, glycerate, and pyruvate), and tricarboxylic acid intermediates (oxaloacetate, fumarate, succinate, and alpha-ketoglutarate) accumulate during dehydration suggesting the importance of these metabolites for the metabolic flux through these pathways during dehydration or in preparation for rehydration (Yobi et al., 2013).

Sugars that accumulate during dehydration in resurrection plants, are proposed to have protective functions such as replacing water on membranes and macromolecules by formation of anhydrous glass (Crowe et al., 1998; Hoekstra et al., 2001), vitrification of the cytoplasm (Leopold and Vertucci, 1986; Vertucci and Farrant, 1995), filling and stabilization of vacuoles (Farrant, 2000), and stabilization of membrane proteins (Hartung et al., 1998; Oliver et al., 1998). A correlation exists between the accumulation of oligosaccharides like raffinose, stachyose, and verbascose with desiccation tolerance in seeds which supports the importance of carbohydrates for acquisition of desiccation tolerance (Horbowicz and Obendorf, 1994; Hoekstra et al., 1997).

### AMINO ACIDS

Protein breakdown and amino acid accumulation are observed in many resurrection plants during dehydration (Tymms and Gaff, 1978; Gaff and McGregor, 1979). While glycine, alanine, and the amino acid-related metabolite quinate are abundant in the fully hydrated tissue of *Selaginella lepidophylla*, amino acids such as glutamine, glutamate, arginine, aspartate, citrulline, asparagine, *N*-6-trimethyllysine, *trans*-4-hydroxyproline, as well as the intermediate metabolites 3-(3-hydroxyphenyl)propionate, the tripeptide ophthalmate (L-Y-glutamyl-L-α-aminobutyrylglycine) are prominent in desiccated tissues (Yobi et al., 2013). Similarly in *Sporobolus stapfianus*, amino acids such as asparagine, arginine, glutamate, glutamine, and the amino acid precursor quinate accumulated during desiccation (Martinelli et al., 2007; Oliver et al., 2011b). These amino acids could function as compatible solutes or as mobile nitrogen reserves for the rehydrating tissues. In *Selaginella lepidophylla* citrulline, a non-proteinogenic amino acid and a structural analog of arginine, is the only amino acid that is more abundant in desiccated tissues than in hydrated tissues. In *C. plantagineum*, amino acid composition did not change significantly during dehydration/rehydration (Bianchi et al., 1992). In a number of species, γ-glutamyl amino acids accumulate during desiccation (Oliver et al., 2011b; Yobi et al., 2013). Addition of glutamyl residues to amino acids prevents their degradation. The amino acid derivative 3-(3-hydroxyphenyl) propionate is more abundant in dehydration and desiccation states in *Selaginella lepidophylla* than in the hydrated tissue. Several aromatic amino acids such as tryptophan and the derivatives acetyltryptophan or phenylalanine which serve as biosynthetic precursors for primary and secondary metabolites accumulate in *Sporobolus stapfianus* and *Selaginella lepidophylla* during dehydration (Oliver et al., 2011b; Yobi et al., 2012). The amino acids phenylalanine and tyrosine accumulate during dehydration in vegetative tissues of *H. rhodopensis* suggesting the activation of the shikimate pathway (Gechev et al., 2013) which can result in the synthesis of antioxidants.

### NUCLEOTIDE METABOLITES

Besides amino acids, the relative abundance of other nitrogen-rich metabolites within the purine and pyrimidine nucleotide pathways is altered during the dehydration/rehydration cycle in *Selaginella lepidophylla* (Yobi et al., 2013). Some nucleotides [e.g., allantoin, 1-methyladenosine, and uridine 5′-monophosphate (UMP)] were more abundant in desiccated tissues than in hydrated tissues. Inosine, a purine nucleoside containing the purine-base hypoxanthine and the sugar D-ribose, accumulated during desiccation in *Selaginella lepidophylla*, whereas 2′-deoxyadenosine was more abundant in fully hydrated *Selaginella lepidophylla*. These results are similar to those observed for *Sporobolus stapfianus*, in which allantoin increased during dehydration (Oliver et al., 2011b).

### LIPIDS

Maintenance of membrane integrity plays a pivotal role in desiccation tolerance (Hoekstra et al., 2001) and therefore, changes in lipid compositions are essential for desiccation tolerance. A detailed lipid analysis was recently reported for *C. plantagineum* and some closely related species (Gasulla et al., 2013). Although the total lipid content remained constant during desiccation, remarkable changes in lipid composition were observed in *C. plantagineum* and *L. brevidens* (Gasulla et al., 2013). An interesting observation was the removal of monogalactosyldiacylglycerol (MGDG) from the thylakoid membranes during desiccation. MGDG was either converted to digalactosyldiacylglycerol or it was hydrolyzed and the resulting diacylglycerol was used for phospholipid and triacylglycerol production (Gasulla et al., 2013). Accumulation of phosphatidylinositol is a specific response observed in desiccation tolerant *C. plantagineum* and *L. brevidens* but not in desiccation sensitive plants suggesting its importance for desiccation tolerance (Gasulla et al., 2013). A common response observed in plants is the increase of phosphatidic acid upon dehydration. Phosphatidic acid is considered to be a signaling molecule which may regulate down-stream processes. The increase in phosphatic acid is due to the activation of phospholipase D under desiccation in *C. plantagineum* (Frank et al., 2000). Lysolipids and fatty acids which are natural products formed by the hydrolysis of phospholipids are accumulated during dehydration. In *Sporobolus stapfianus*, accumulation of lysolipids suggests the scope for minimal damage to lipid membranes during dehydration (Oliver et al., 2011b). In *Selaginella lepidophylla* out of the 32 lipid metabolites, only choline phosphate accumulated in response to dehydration. Membranes are protected during dehydration in *Selaginella lepidophylla* as polyunsaturated fatty acids and markers of lipoxygenase activity increased (Yobi et al., 2013). A decrease in the relative amounts of various unsaturated lipids upon dehydration has been reported for the desiccation tolerant *Sporobolus stapfianus* (Quartacci et al., 1997) and *Ramonda serbica* (Quartacci et al., 2002). These alterations in unsaturated fatty acid concentrations are supposed to contribute to membrane fluidity to allow for recovery after dehydration (Upchurch, 2008).

### POLYAMINES

Polyamines are low molecular weight polycationic compounds involved in cellular processes, such as membrane stabilization, enzyme activity modulation, plant growth and development, nitrogen assimilation, and respiratory metabolism (Alcázar et al., 2010). Polyamines are able to bind to negatively charged molecules like DNA, proteins, or membrane phospholipids and thus are able to protect these macromolecules. A positive correlation exists between the abundance of polyamine levels and stress tolerance in plants (Alcázar et al., 2010). In agreement with this, putrescine, spermidine, and spermine were found to be at higher levels after drought treatment in *C. plantagineum* than in *Arabidopsis* putrescine levels along with spermidine and spermine increased after 96 h of dehydration in *C. plantagineum* (Alcázar et al., 2011). This implies an early drought response phenomenon in *C. plantagineum* and indicates that stimulation in spermidine and spermine biosynthesis occurs with concomitant reduction of putrescine precursor levels. It suggests a metabolic canalization of putrescine to spermine in *C. plantagineum*. In drought sensitive *Arabidopsis*, although the putrescine to spermine canalization occurs under dehydration, the spermidine and spermine levels do not increase and this is due to conversion of spermine to putrescine thereby forming a polyamine recycling-loop during drought acclimation (Alcázar et al., 2011).

### ANTIOXIDANTS

Most of the cellular damage occurs through the activity of ROS (Smirnoff, 1993). ROS are particularly prevalent during desiccation of photosynthetic tissues because chlorophyll retains the ability to transfer electrons while carbon fixation does not take place. Under light conditions a flow of electrons and energy is passed from chlorophyll molecules to ground state oxygen thus generating singlet oxygen. To detoxify the ROS plant cells contain a wide array of reactive oxygen scavenging antioxidant enzymes (superoxide dismutase, catalase, ascorbate peroxidase, etc.) along with low molecular weight antioxidants. Both desiccation sensitive and tolerant plants up-regulate antioxidant synthesis to detoxify ROS upon dehydration. A major difference between resurrection plants and desiccation sensitive plants appears to be in the ability to maintain the antioxidant levels during the later stages of desiccation where oxidative stress prevails (Mowla et al., 2002; Illing et al., 2005). The importance of low molecular weight antioxidants (ascorbate and GSH), tocopherols, phenolic acids, or polyphenols (galloylquinic acid) has been deduced from metabolic analyzes (Kranner et al., 2002; Moore et al., 2005). Ascorbate–GSH cycle metabolites are often elevated during desiccation in resurrection plants (Navari-Izzo et al., 1997; Jiang et al., 2007). *M. flabellifolia* contains large amounts of antioxidants in the hydrated state suggesting that the antioxidant-based protection mechanisms are constitutively expressed. However, desiccation still leads to increased levels of reduced GSH and oxidized GSH (GSSG; Kranner et al., 2002). Unlike GSH, both ascorbate and dehydroascorbate levels decreased during desiccation in *M. flabellifolia*, and were completely depleted after four months of desiccation, which compromised the survival mechanism (Kranner et al., 2002). A similar pattern is observed in *H. rhodopensis* leaves where the total GSH levels and the GSSG/GSH ratio increased upon desiccation (Djilianov et al., 2011). In *Sporobolus stapfianus*, γ-glutamyl dipeptides that are involved in GSH recycling via the γ-glutamyl cycle and GSSG were increased during desiccation (Oliver et al., 2011b). In *T. ruralis*, the most abundant dehydration-responsive γ-glutamyl dipeptides are γ-glutamylisoleucine, γ-glutamylleucine, γ-glutamylvaline, and γ-glutamylphenylalanine.

Apart from common antioxidants like GSH or ascorbate, polyphenols such as 3,4,5-tri-*O*-galloylquinic acid accumulated in *M. flabellifolia*, and seed-associated antioxidants, e.g., 1-Cys peroxiredoxin and metallothioneins occurred in *X. viscosa* (Ndima et al., 2001; Mowla et al., 2002; Moore et al., 2005). Polyphenols are powerful detoxifiers of ROS and are present in some resurrection plants (Veljovic-Jovanovic et al., 2008). In *Selaginella lepidophylla*, phenolics (e.g., caffeate), flavonols (e.g., apigenin and naringenin), and phenylpropanoids (e.g., coniferyl alcohol) accumulated in desiccated tissues (Yobi et al., 2013). In *H. rhodopensis*, phenols reached around 15–20% of the total dry weight of the desiccated plant. In contrast, the phenolic acids in *Ramonda serbica* decreased under desiccation and increased upon rehydration (Sgherri et al., 2004; Veljovic-Jovanovic et al., 2008) suggesting the operation of different mechanisms. In *Sporobolus stapfianus*, both alpha and beta-tocopherols are induced during desiccation whereas they are at low levels in *M. flabellifolia* and *Selaginella lepidophylla* (Kranner et al., 2002; Oliver et al., 2011b; Yobi et al., 2013). The examples described demonstrate diversity in the antioxidants in resurrection plants. This might be determined by the environmental factors at the habitat of the species.

## COMPARATIVE OMICS STUDIES BETWEEN DESICCATION TOLERANT AND SENSITIVE SPECIES

Two types of comparisons can be performed to analyze the changes in gene expression, protein, or metabolite levels across species. The ancestor-descendent comparison depends on the reconstruction of the evolutionary history of the gene and its association with desiccation tolerance. The sister group comparison can be made for two closely related species that differ in desiccation tolerance. Both approaches have been applied. The aim of these comparative approaches is to identify components which are linked with desiccation tolerance. Illing et al. (2005) compared the expression profiles of different genes in vegetative tissues of desiccation tolerant *X. viscosa* with *Arabidopsis* seed specific genes from available expression data. The expression profiles of various LEA genes and antioxidant genes that are induced during desiccation in *X. viscosa* vegetative tissues are also induced during seed development in *Arabidopsis* suggesting similarities between the response to desiccation in the resurrection plant and in seeds. In another study, a comparative analysis of antioxidant gene expression was performed between the two desiccation tolerant species *C. plantagineum* and *L. brevidens* and the desiccation sensitive species *L. subracemosa*, all belonging to the same family. Antioxidant genes were either constitutively expressed or up-regulated during desiccation and rehydration in desiccation tolerant species but down-regulated in the desiccation sensitive *L. subracemosa* (Dinakar and Bartels, 2012). Comparative lipid profiles in the same species identified phosphoinositol as a compound associated with desiccation tolerance (Gasulla et al., 2013; see also Section “Lipids”). Phosphoinositol could replace water due to the hydroxyl groups and therefore could contribute to maintain structures of macromolecules. Species specific sequencing of EST clones from the desiccation tolerant *Selaginella lepidophylla* and the desiccation sensitive *Selaginella moellendorffii* identified ESTs associated with desiccation tolerance (Iturriaga et al., 2006). ESTs for transporters, cell structure, molecular chaperones, and LEA genes were more abundant in *Selaginella lepidophylla* than in *Selaginella moellendorffii*.

Comparative metabolomic analysis between desiccation tolerant and sensitive species has been performed in *Sporobolus stapfianus* vs *Sporobolus pyramidalis* and *Selaginella lepidophylla* vs *Selaginella moellendorffii* respectively (Oliver et al., 2011b; Yobi et al., 2012). Metabolomic comparison between desiccation tolerant *Sporobolus stapfianus* and desiccation sensitive *Sporobolus pyramidalis* demonstrated that *Sporobolus stapfianus* is metabolically primed for desiccation by accumulating osmoregulatory metabolites (Oliver et al., 2011b). *Sporobolus stapfianus* has higher levels of osmolytes, nitrogen source compounds and lower concentrations of compounds related to energy metabolism and growth than *Sporobolus pyramidalis* (Oliver et al., 2011b). Several of the polyols are also more abundant in *Sporobolus stapfianus* under hydrated conditions than in *Sporobolus pyramidalis* suggesting that constitutive expression of these polyols is a strategy to combat oxidative stress (Oliver et al., 2011b). Like *Sporobolus stapfianus*, desiccation tolerant *Selaginella lepidophylla* retained higher amounts of sucrose, mono- and polysaccharides, and sugar alcohols (sorbitol, xylitol) than desiccation sensitive *Selaginella moellendorffii* (Yobi et al., 2012). Another common feature between *Sporobolus stapfianus* and *Selaginella lepidophylla* is the abundancy of the amino acids alanine and leucine in hydrated conditions and their increase during dehydration (Oliver et al., 2011b; Yobi et al., 2012). However, both species differ in the accumulation of other amino acids (asparagine, aspartate, arginine, and glutamate), which are higher in *Sporobolus stapfianus* than in *Sporobolus pyramidalis* under hydrated conditions, and *vice versa* in *Selaginella* (Oliver et al., 2011b; Yobi et al., 2013). The desiccation tolerant *Selaginella lepidophylla* accumulates more γ-glutamyl amino acids during dehydration than *Selaginella moellendorffii* (Yobi et al., 2013). This feature is also observed in desiccating *T. ruralis* and *Sporobolus stapfianus* which suggests that it has been conserved during evolution (Oliver et al., 2011b). Some differences between *Sporobolus* and *Selaginella* could also be related to the difference in the life cycle of the plants.

## INTEGRATING THE OMICS RESPONSES AND NEW INSIGHTS IN UNDERSTANDING THE MECHANISM OF DESICCATION TOLERANCE

**FIGURE 1:**
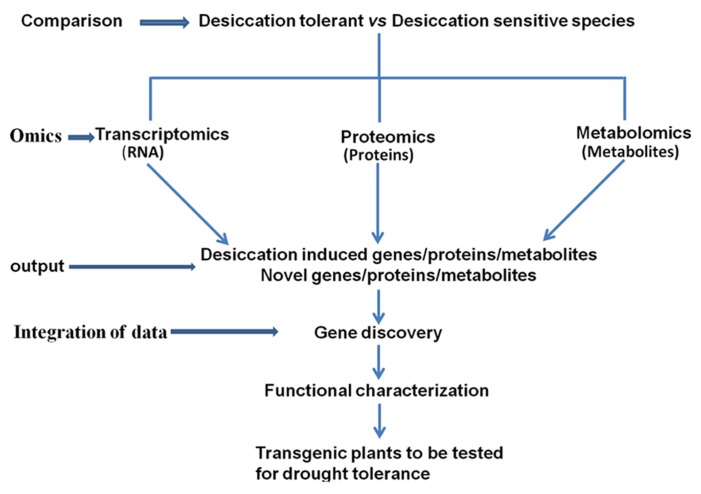
**Comparative studies of desiccation tolerant and sensitive plants using omics approaches and integration of the data to identify target genes for generating drought tolerant plants**.

Integration of the data obtained from transcriptomic, proteomic, and metabolomic studies in resurrection plants is important for decoding the desiccation tolerance mechanisms (**Figure 1**). The summary of the omics studies and sister group comparisons is shown in **Table 1**. Eventually the knowledge on pathways leading to desiccation tolerance could be used for improving drought tolerance in crops (**Figure 1**). Evidence from omics studies in resurrection plants suggests that some protective strategies are constitutively active in resurrection plants and do not require induction like in dehydration sensitive plants. This indicates preparedness of the plant for dehydration stress (Gechev et al., 2013). The comparative metabolomic analysis of desiccation tolerant and sensitive *Sporobolus* and *Selaginella* species demonstrates that sensitive plants loose water more rapidly during dehydration than desiccation tolerant plants suggesting that slowing down the rate of water loss might allow more time for the synthesis of protective molecules and important for acquisition of desiccation tolerance (Oliver et al., 2011b; Yobi et al., 2012).

The common observation from the metabolomic analysis is the abundance of amino acids, sugar alcohols, and other compatible solutes in hydrated vegetative tissues of desiccation tolerant plants thereby reducing the speed of water loss from the plants allowing the plant to synthesize compounds required for desiccation tolerance. Comparative omics studies have aided in differentiating the responses of tolerant and sensitive species thereby giving an overview of the molecular processes that contribute to desiccation tolerance (Rodriguez et al., 2010; Gechev et al., 2013).

Reduction in photosynthesis, accumulation of LEA proteins and carbohydrates, increased antioxidants along with increased enzymatic activities are commonly observed in taxonomically distant resurrection plants. Both desiccation sensitive and desiccation tolerant plants share mechanisms of drought perception and responses such as induction of LEA proteins, heat shock genes, or adjustment of carbohydrate metabolism. However, quantitatively levels seem to vary. In addition, some resurrection plants seem to express unique metabolites such as the eight-carbon sugar octulose, and the CDT1 gene in *C. plantagineum*, or the phenolic antioxidant 3,4,5-tri-*O*-galloylquinic acid in *M. flabellifolia*.

## CONCLUSIONS AND FUTURE PERSPECTIVES

**FIGURE 2:**
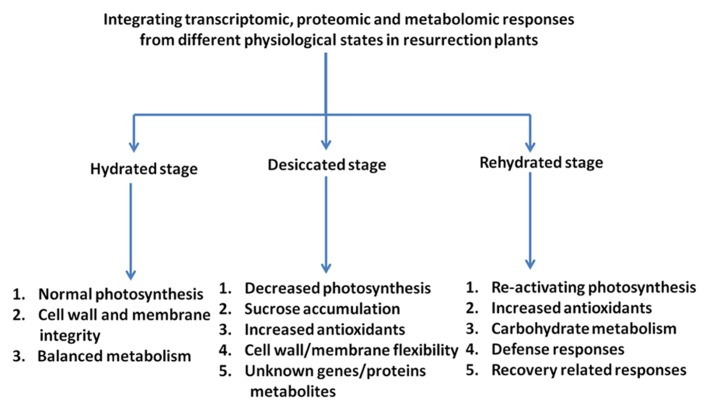
**Diagrammatic representation of the responses of resurrection plants to desiccation/rehydration as analyzed by omics approaches.** Indicated are the main classes of transcripts/metabolites characteristic for the different stages of the dehydration/rehydration cycle.

Elucidation of the molecular mechanism of desiccation tolerance in resurrection plants is difficult due to the lack of genetic approaches and due to the complexity of the phenomenon. However, with the realization of omics technologies it is possible to provide a comprehensive description of changes on the transcript, proteome, and metabolome levels during a dehydration/rehydration cycle (**Figure 2**). The combination of the data should lead to a systems biology approach and identify target genes and critical metabolic pathways. The studies carried out with resurrection species to date show that similarities exist in dehydration-induced gene expression as well as in protein and metabolite accumulation in resurrection plants pointing toward the presence of common mechanisms of desiccation tolerance. It is proposed that the tolerance to desiccation in vascular resurrection plants is due to the combinatorial effect of accumulation of high levels of antioxidants together with various osmoprotective compounds such as sugars, in particular sucrose and raffinose, and hydrophilic proteins, most prominently LEA proteins. The mechanisms in vascular resurrection plants appear to be different from lower plants like mosses. Desiccation tolerance mechanisms in seeds and vascular plants share many components, but they differ in the regulation of accumulation of components (Farrant and Moore, 2011). In seeds, the expression of desiccation tolerance is strictly developmentally regulated while it is expressed throughout most of the life cycle in resurrection plants. Therefore, the elucidation and intensive study of regulatory expression networks should be subject of future research. There are a number of questions which have hardly been experimentally addressed like which components allow the cells to shrink during desiccation and expand again during rehydration or how are thylakoid membranes functionally re-assembled during rehydration. These processes probably require very specific membrane and cell wall compositions.

A remarkable finding in the transcriptome studies in *C. plantagineum* and *H. rhodopensis* is the fact that a high number of transcripts exist which could not be assigned to known sequences from other species. This suggests that some parts of the transcript coding genome has evolved very recently leading to unique genes possibly by genome rearrangements, genome duplication, and activity of transposable elements and/or retro-elements. It appears from studies in *C. plantagineum* that not only protein coding sequences are unique but also non-protein coding RNAs, which are present in many copies in the genome and are abundantly expressed during dehydration (Furini et al., 1997; Giarola and Bartels unpublished). Genome plasticity may be required for extreme adaptation, as it was already observed for genomes of halophytes (Bartels and Dinakar, 2013). The studies on these orphan genes are essential for understanding mechanisms of desiccation tolerance.

## Conflict of Interest Statement

The authors declare that the research was conducted in the absence of any commercial or financial relationships that could be construed as a potential conflict of interest.
